# Epstein-Barr Virus Zta Upregulates Matrix Metalloproteinases 3 and 9 That Synergistically Promote Cell Invasion In Vitro

**DOI:** 10.1371/journal.pone.0056121

**Published:** 2013-02-07

**Authors:** Yu-Yan Lan, Tzu-Hao Yeh, Wei-Hung Lin, Shih-Yi Wu, Hsiao-Ching Lai, Fang-Hsin Chang, Kenzo Takada, Yao Chang

**Affiliations:** 1 National Institute of Infectious Diseases and Vaccinology, National Health Research Institutes, Tainan, Taiwan; 2 Graduate Institute of Basic Medical Sciences, Medical College and Hospital, National Cheng Kung University, Tainan, Taiwan; 3 Department of Microbiology and Immunology, Medical College and Hospital, National Cheng Kung University, Tainan, Taiwan; 4 Department of Tumor Virology, Institute for Genetic Medicine, Hokkaido University, Sapporo, Japan; The Chinese University of Hong Kong, Hong Kong

## Abstract

Zta is a lytic transactivator of Epstein-Barr virus (EBV) and has been shown to promote migration and invasion of epithelial cells. Although previous studies indicate that Zta induces expression of matrix metalloproteinase (MMP) 9 and MMP1, direct evidence linking the MMPs to Zta-induced cell migration and invasion is still lacking. Here we performed a series of *in vitro* studies to re-examine the expression profile and biologic functions of Zta-induced MMPs in epithelial cells derived from nasopharyngeal carcinoma. We found that, in addition to MMP9, MMP3 was a new target gene upregulated by Zta. Ectopic Zta expression in EBV-negative cells increased both mRNA and protein production of MMP3. Endogenous Zta also contributed to induction of MMP3 expression, migration and invasion of EBV-infected cells. Zta activated the MMP3 promoter through three AP-1 elements, and its DNA-binding domain was required for the promoter binding and MMP3 induction. We further tested the effects of MMP3 and MMP9 on cell motility and invasiveness *in vitro*. Zta-promoted cell migration required MMP3 but not MMP9. On the other hand, both MMP3 and MMP9 were essential for Zta-induced cell invasion, and co-expression of the two MMPs synergistically increased cell invasiveness. Therefore, this study provides integrated evidence demonstrating that, at least in the *in vitro* cell models, Zta drives cell migration and invasion through MMPs.

## Introduction

Epstein-Barr virus (EBV) is a human herpesvirus which infects both lymphoid and epithelial cells and contributes to pathogenesis of several lymphomas and carcinomas. Nasopharyngeal carcinoma (NPC) is an epithelial cancer endemic in southern China, southeast Asia, the Arctic, and North Africa [1]. In the endemic areas, the strong association between NPC and EBV is supported by prevalent detection of viral genomes, transcripts, and antigens in the tumor specimens [2]. Although EBV majorly adopts latent infection in NPC tumors, a small subset of the tumor cells undergo abortive lytic infection where some immediate early or early viral genes are expressed but late lytic transcripts are rarely detected [3]–[5]. Some clues suggest that EBV reactivation into the lytic cycle is linked to development or progression of NPC. Elevated antibody titers in sera against EBV lytic antigens predict a high risk of NPC [6] and are also correlated with advanced clinical stage, poor prognosis, or tumor recurrence of NPC [7]–[9]. Meanwhile, some environmental or dietary factors associated with a high incidence of NPC act as not only carcinogens but also potent inducers of the viral lytic cycle [10], [11]. Recent studies have also suggested that EBV reactivation and certain lytic proteins enhance genome instability of NPC cells [12], [13].

Another link between lytic EBV infection and NPC comes from the potential contribution of a viral lytic protein Zta to NPC metastasis. Zta, also named BZLF1, is a unique member of the basic leucine-zipper (b-Zip) transcription factors and functions as an essential transactivator for the switch from EBV latency to the lytic cycle [14], [15]. It forms a homodimer and binds to its target promoters through the DNA elements that are identical or similar to the binding sites for other cellular b-Zip proteins such as AP-1 or C/EBP [16]. Through the promoter binding, Zta regulates transcription of not only viral lytic genes but also some cellular genes [17]–[20]. Previous studies indicate that anti-Zta antibodies are increased in NPC patients [21] and the patients with higher titers of anti-Zta antibodies have a poorer clinical outcome owing to high incidence of tumor metastasis [9]. Notably, an immunohistochemical study shows that positive detection of Zta protein in tumor cells is correlated with advanced NPC metastasis to neck lymph nodes [4]. The potential of Zta to promote metastasis is further supported by an *in vitro* study showing that stable Zta expression in a keratinocyte cell line enhances cell motility and invasiveness in a collagen gel [22].

How Zta promotes cell migration and invasion is largely unknown. Two previous studies suggest that it may involve induction of matrix metalloproteinases (MMPs), a family of zinc-dependent proteolytic enzymes associated with multiple processes of cancer progression, including cell growth, migration, invasion, and angiogenesis [23], [24]. Zta upregulates MMP9 in a cervical carcinoma cell line but the biologic effects of Zta-induced MMP9 on this cell line have not been tested previously [4]. On the other hand, MMP1 is induced by Zta in a keratinocyte cell line and essential for survival of the cells growing in a collagen gel, while the contribution of MMP1 to cell migration or invasion has not been shown [22]. These two studies indicate that Zta upregulates different MMPs probably in a cell-dependent manner. However, we are not sure whether and what Zta-induced MMPs functionally contribute to cell motility or invasiveness.

For two specific aims, we used a series of *in vitro* studies to re-examine the expression profile and biologic functions of Zta-induced MMPs in epithelial cells derived from NPC. The first aim is to test whether multiple MMPs can be co-induced by Zta in NPC cells. Since promoters of some MMP genes contain similar *cis*-regulatory elements, these MMPs are likely to be co-regulated. For example, MMP9, MMP14, and MMP15 are regulated by the Rb-E2F pathway and co-expressed in non-small cell lung cancer [25], while MMP2 and MMP9 are co-induced by some other mechanisms [26], [27]. The second aim of this study is to prove that MMPs functionally contribute to Zta-induced migration and invasion of NPC cells *in vitro*. Since various MMPs exert distinct or overlapped functions [24], we speculated that co-induction of multiple MMPs may be required for the full biologic effects of Zta. Indeed, we found that MMP3 and MMP9 were co-upregulated by Zta through a similar mechanism. Furthermore, the Zta-induced MMP3 and MMP9 differentially affected cell migration but synergistically contributed to cell invasion. Therefore, our results reveal an underlying mechanism of Zta-induced cell migration and invasion.

## Materials and Methods

### Cell Culture, Drug Treatment, and Induction of EBV Reactivation

EBV-negative HONE-1 is an epithelial cell line derived from NPC tumors [28]. The doxycycline-inducible, Zta-expressing HONE-tetonZ cells were previously generated from HONE-1 cells [29]. The doxycycline-inducible, Rta-expressing TW01-tetER cells were generated from NPC-TW01 cells [30], and the EBV-infected TW01-ERGV cells were established by *in vitro* conversion of TW01-tetER cells with a recombinant EBV expressing green fluorescence protein [31]. SCC15 is a cell line derived from oral squamous cell carcinoma [32], and AGS is a cell line derived from gastric carcinoma [33]. All the epithelial cell lines were cultured in RPMI 1640 medium supplemented with 10% fetal bovine serum (HyClone, UT, USA) at 37°C with 5% CO_2_. MMP3 activity was inhibited by treatment of cells with 10 µM MMP3 inhibitor II (Calbiochem, CA, USA) for 48 h, while MMP9 activity was inhibited by treatment with 10 µM MMP9 inhibitor I (Calbiochem) for 48 h. For induction of Zta expression in HONE-tetonZ cells, the cells were treated with doxycycline (1 µg/ml) (Sigma, MO, USA) for 72 h. For induction of Rta expression and EBV reactivation in TW01-ERGV cells, the cells were treated with doxycycline (50 ng/ml) for 48 h.

### Plasmids, siRNAs, and Cell Transfection

DNA plasmids expressing wild-type Zta or its DNA binding-defective mutants have been used previously [18], [34]. The pGL2-based reporter plasmids driven by the wild-type MMP3 promoter (-2290 to +10) or its derivatives with mutations at various AP-1 sites have also been described in a previous study [35]. Plasmids expressing human MMP3 and MMP9 were purchased from Origene (Rockville, MD, USA). All synthetic siRNAs in this study were purchased from Invitrogen (Carlsbad, CA, USA), including two siRNAs targeted against Zta (siZta-1 5′- UUCAGAAGUCGAGUUUGGGUCCAUC-3 and siZta-2 5′- UUCUAGUUCAGAAUCGCAUUCCUCC-3′), two against MMP3 (siMMP3-1 5′-UCAACAAUUAAGCCAGCUGUUACUC-3′ and siMMP3-2 5′-UUCCUUAUCAGAAAUGGCUGCAUCG-3′), two against MMP9 (siMMP9-1 5′-AAGGUUUGGAAUCUGCCCAGGUCUG-3′ and siMMP9-2 5′-AAUACAGCUGGUUCCCAAUCUCCGC-3′), and a control siRNA with comparable GC content. Single plasmid transfection and dual plasmid/siRNA transfection were performed as described previously [36] by using Lipofectamine 2000 reagent (Invitrogen). At 4 h posttransfection, cells were washed and cultured in serum-free medium for further experiments.

### MMP Antibody Array and Enzyme-linked Immunosorbent Assay (ELISA)

Expression profiles of MMPs in the cell culture supernatants were analyzed by using a human MMP antibody array (RayBiotech, GA, USA) according to the protocol provided in the kit. MMP3 in the cell culture supernatants were quantified by using the DuoSet ELISA kit (R&D Systems, MN, USA) according to the manufacturer’s instruction.

### Antibodies and Immunoblotting Assay

Primary antibodies used in the immunoblotting assay included mouse monoclonal antibodies recognizing Zta (4F10), Rta (467), EA-D (88A9), and β-actin (C4; purchased from Chemicon, Billerica, MA, USA). Extraction of cellular proteins and the subsequent analysis by electrophoresis and the immunoblotting assay were carried out as described previously [18].

### RNA Extraction and Quantitative Reverse Transcription (RT)-PCR

Extraction of cellular RNA and synthesis of cDNA were carried out as described previously [36]. Real-time PCR analysis for quantification of MMP3 cDNA was performed by using LightCycler reagents and the compatible detection system (Roche, CT, USA). PCR primers for detecting MMP3 were 5′-GCAGTTTGCTCAGCCTATCC-3′ and 5′-TTTCTCCTAACAAACTGTTTCACATC-3′, and the locked nucleic acid-based probe was 5′- GGATGGAG-3′. Primers for detecting an internal reference, TATA box-binding protein, were 5′-GCTGGCCCATAGTGATCTTT-3′ and 5′-TCCTTGGGTTATCTTCACACG-3′, and the probe was 5′-CCCAGCAG-3′. Duplicated experiments were carried out and the relative MMP3 mRNA levels were calculated by using the LightCycler software (Roche).

### Reporter Gene Assay and Chromatin Immunoprecipitation (ChIP) Assay

Firefly luciferase activity reflecting the promoter activity of reporter plasmids was detected as described previously [18] by using a Bright-Glo assay kit (Promega, WI, USA). Recruitment of Zta to the target promoter was detected by using the ChIP assay as described in our previous study [37]. Zta was immunoprecipitated by using a mouse monoclonal antibody AZ-69 (Argene, Varilhes, France). DNA fragment of the MMP3 promoter in the immunoprecipitants were quantified by real-time PCR using the forward primer 5′-AATTTGGAATGTTTGGAAATGG-3′, the reverse primer 5′-GCTTGACTCATCCTTGCTTTC-3′, and the probe 5′- CTGCTGCC-3′. The relative amounts of target promoter DNA in the immunoprecipitants were calculated and presented as the “percentage of input”.

### Gelatin Zymography

MMP9 and MMP2 in the cell culture supernatants were detected by gelatin zymography as described previously [36]. In brief, samples were electrophoresed on an 8% polyacrylamide gel containing gelatin (1 mg/ml) under nonreducing conditions. After electrophoresis, MMP9 and MMP2 in the gels were renatured and reacted with their substrate gelatin. Subsequent gel staining with Coomassie blue R250 visualized the transparent bands of 92-kDa MMP9 and 72-kDa MMP2.

### Transwell Cell Migration Assay and Matrigel Invasion Assay

Transwells (6.5-mm diameter and 8-µm pore size; Corning, MA, USA) were used for the cell migration assay, while Biocoat Matrigel invasion chambers (Becton Dickinson, NJ, USA) coated with extracellular matrix (ECM) were used for the cell invasion assay. Both assays were carried out in 24-well plates in similar ways. Cells suspended in serum-free RPMI 1640 medium were plated onto the upper chambers (25000 cells/well) of transwells or Matrigel chambers, and the lower chambers contained 10% fetal bovine serum as the chemoattractant. After incubation at 37°C in 5% CO_2_ for 24 h, the cells remaining on the upper chambers were removed by a cotton swab, and the cells migrating or invading to the underside of the membrane were fixed and stained with Giemsa dye. In each chamber, the migrating or invading cells were counted from five randomly-chosen microscopic fields and the average cell numbers per field were calculated.

## Results

### Zta Upregulates MMP3 and MMP9

**Figure 1:**
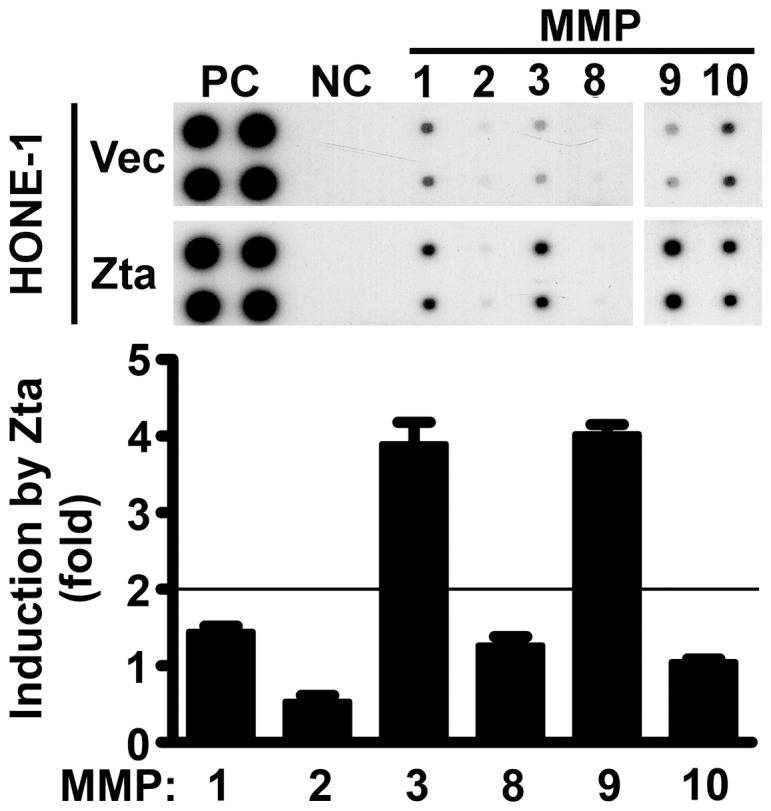
MMP3 and MMP9 are upregulated by Zta. HONE-1 cells were transfected with a vector plasmid (Vec) or a Zta-expressing plasmid (Zta). In the upper panel, the profiles of MMP production in the cell culture supernatants were analyzed by using an antibody array, where the positive control (PC) and the negative control (NC) were included. In the lower panel, Zta-mediated induction of each MMP was quantified as folds of induction compared with the vector control. In this analysis, only MMP3 and MMP9 were induced more than two folds by Zta.

First we used an MMP antibody array to analyze the conditioned medium of an EBV-negative NPC cell line, HONE-1, with or without ectopic expression of Zta. Compared with the vector transfectants, Zta-transfected cells showed more than twofold increase of production of MMP3 and MMP9 (Fig. 1). MMP1, which can be induced by Zta in a keratinocyte cell line [22], was only slightly (1.5 fold) upregulated by Zta in HONE-1 cells, suggestion that Zta-mediated regulation of MMP1 is cell-dependent. Zta-mediated induction of MMP9 and its underlying mechanism have been studied previously [4], but the effect of Zta on MMP3 expression has not been reported before. Therefore, we focused on the induction of MMP3 in following experiments.

### Zta Induces MMP3 Expression

**Figure 2:**
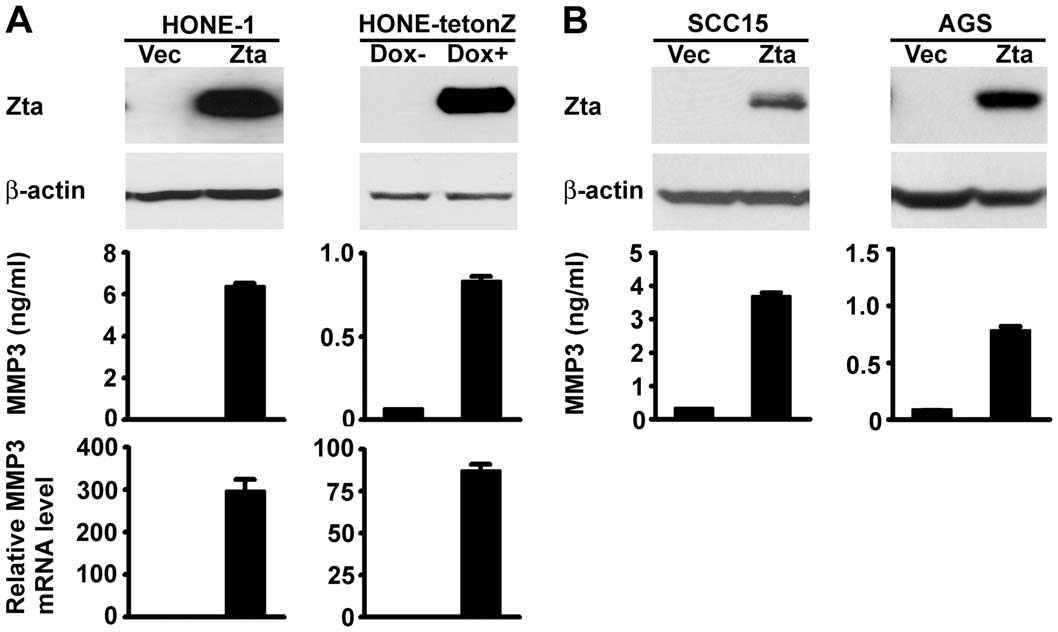
Zta induces MMP3 expression. (**A**) HONE-1 cells were transfected with a vector plasmid (Vec) or a Zta-expressing plasmid, and HONE-tetonZ cells were treated with (+) or without (-) doxycycline (Dox). In the upper panel, protein expression of Zta and β-actin was examined by an immunoblotting assay. In the middle panel, concentrations of MMP3 protein in the cell culture supernatants were quantified by using ELISA. In the lower panel, the expression levels of MMP3 mRNA were measured by quantitative RT-PCR. (**B**) SCC15 and AGS cells were transfected with a vector plasmid (Vec) or a Zta-expressing plasmid. Expression of Zta and β-actin was examined by an immunoblotting assay, and concentrations of MMP3 protein in the cell culture supernatants were quantified by using ELISA.

To confirm Zta-mediated MMP3 induction, the mRNA and protein levels of MMP3 were further examined by using quantitative RT-PCR and ELISA, respectively. Transient Zta expression in EBV-negative NPC cells was carried out either through plasmid transfection into HONE-1 cells or by using a doxycycline-inducible, Zta-expressing derivative, HONE-tetonZ [29]. In both cell models, ectopic expression of Zta significantly increased intracellular MMP3 mRNA and also the secreted MMP3 protein in the conditioned medium (Fig. 2A). Zta was also able to induce MMP3 production from two EBV-negative, non-NPC epithelial cell lines, SCC15 and AGS (Fig. 2B), indicating that Zta-mediated induction of MMP3 is not restricted to NPC cells.

### Endogenous Zta Contributes to Induction of MMP3 Expression, Migration and Invasion of EBV-infected Cells

**Figure 3:**
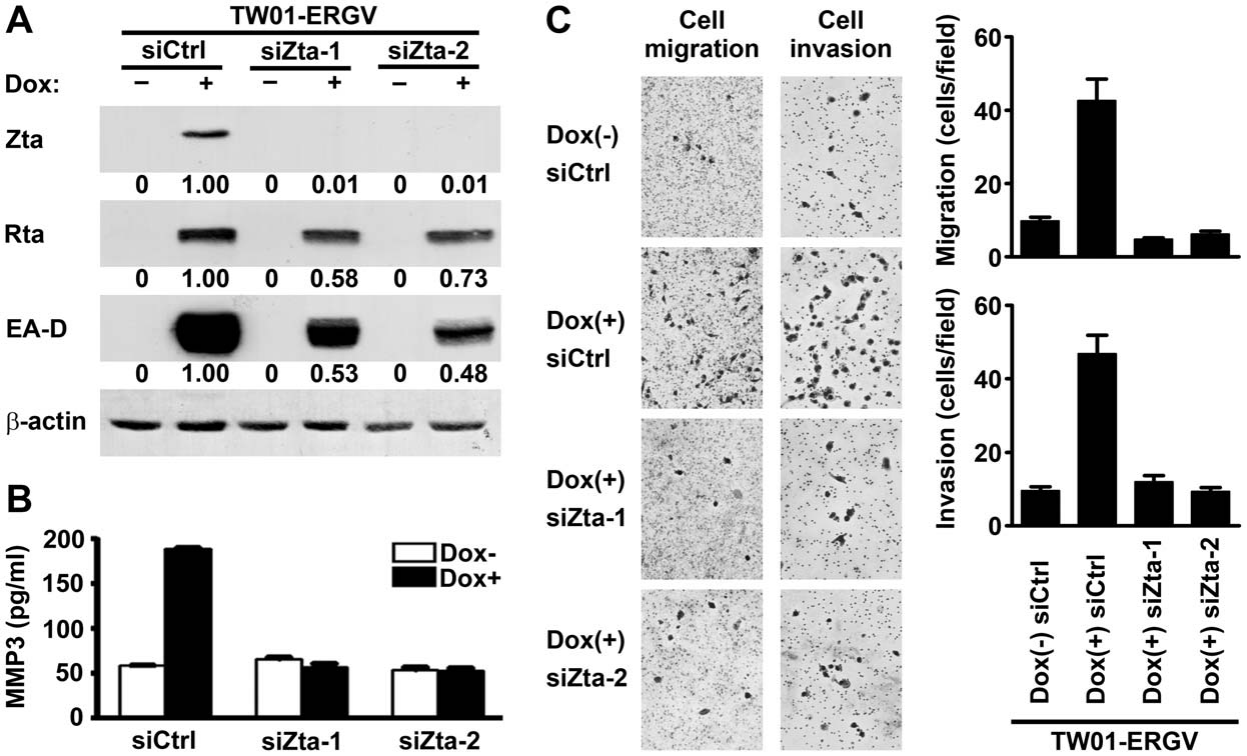
Endogenous Zta contributes to induction of MMP3 expression, migration and invasion of EBV-infected cells. EBV-infected TW01-ERGV cells were transfected with control siRNA (siCtrl) or Zta-targeted siRNAs (siZta-1 and siZta-2) in the presence (+) or absence (-) of doxycycline (Dox) treatment. (**A**) EBV lytic proteins (Zta, Rta and EA-D) and cellular β-actin were detected by using an immunoblotting assay. The relative expression levels of individual viral proteins were quantified by normalization with their corresponding β-actin, and the expression level in the control siRNA-transfected, doxycycline -treated cells was set as 1.00. (**B**) MMP3 concentrations in the cell culture supernatants were measured by using ELISA. (**C**) Cell migration was examined by using a transwell migration assay, and cell invasion in ECM was examined by using a Matrigel invasion assay. Shown are the microscopic observations of migrating or invading cells and the average numbers of the cells per field.

Next we wondered whether endogenous Zta expressed in EBV-infected cells can be also involved in upregulation of MMP3. We examined an EBV-infected NPC cell line, TW01-ERGV, where doxycycline treatment drives expression of a lytic transactivator Rta, resulting in induction of endogenous Zta and its downstream lytic genes [38]. After doxycycline treatment, TW01-ERGV cells expressed Rta, Zta and a downstream lytic protein EA-D/BMRF1 (Fig. 3A), and meanwhile their production of MMP3 was also increased (Fig. 3B). To clarify the role of endogenous Zta, we transfected the cells with Zta-targeted siRNAs, which inhibited expression of endogenous Zta and Zta-induced Rta [20] while did not affect expression of doxycycline-induced exogenous Rta. As expected, transfection with the Zta siRNAs completely blocked Zta expression but only partially reduced Rta expression (Fig. 3A). Since EA-D expression is regulated by both Zta and Rta [17], knockdown of endogenous Zta reduced half the level of EA-D (Fig. 3A). Notably, knockdown of endogenous Zta abolished the induction of MMP3 (Fig. 3B), supporting that Zta is important for regulation of MMP3 expression in the context of EBV-infected cells. We further tested the effect of endogenous Zta on cell migration and invasion. Cell motility was examined by using a transwell migration assay, and cell invasiveness in ECM was examined by using a Matrigel invasion assay. Doxycycline-induced EBV reactivation enhanced migration and invasion of TW01-ERGV cells, and both the effects were inhibited when endogenous Zta was knocked down by siRNAs (Fig. 3C). This result supports that, during lytic EBV infection, endogenous Zta is an important trigger of cell migration and invasion. Meanwhile we do not rule out the possibility that downstream lytic proteins induced by Zta may be also involved in the biologic effects.

### Zta-induced Activation of the MMP3 Promoter Requires Three AP-1 Elements

**Figure 4:**
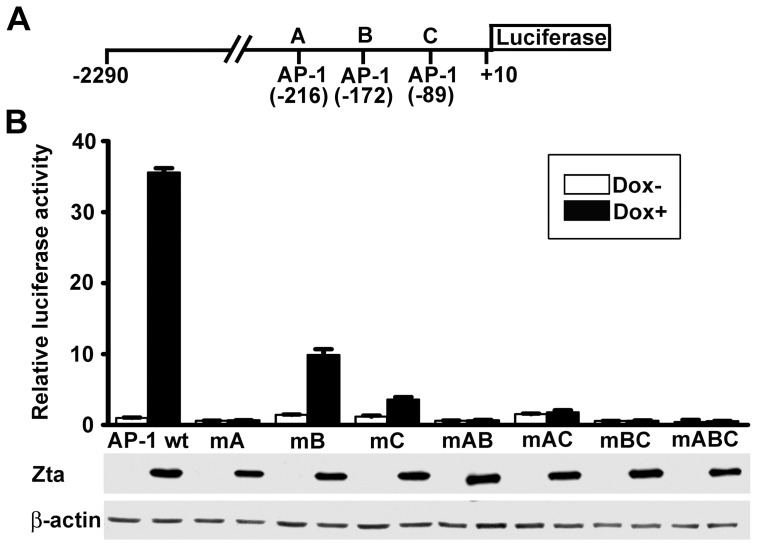
Zta-induced activation of the MMP3 promoter requires three AP-1 elements. (**A**) The MMP3 promoter used in the reporter gene assay is illustrated schematically. Three AP-1 elements at −216, −172, and −89 of the promoter are indicated and labeled as A, B, and C, respectively. (**B**) The reporter plasmids driven by the wild-type (wt) MMP3 promoter or its AP-1-mutated derivatives (mA, mB, mC, mAB, mBC, mAC, and mABC) were transfected into HONE-tetonZ cells in the presence (black bars) or absence (white bars) of doxycycline induction. The promoter activity was examined by using a luciferase assay. Expression of Zta and β-actin was examined by an immunoblotting assay.

A previous study shows that Zta transactivates the MMP9 promoter and an AP-1 site in the promoter is essential for the transactivation [4]. Since the MMP3 promoter contains three AP-1 elements [35], as illustrated in Figure 4A, we examined whether Zta can also regulate activity of the MMP3 promoter. Using doxycycline-inducible, Zta-expressing HONE-tetonZ cells, we found that Zta significantly activated the wild-type MMP3 promoter (−2290 to +10) in the reporter gene assay (Fig. 4B). Accordingly, the MMP3 promoter constructs with various mutations at AP-1 sites were further tested. Disruption of any of the three AP-1 elements at −216, −172 and −89 profoundly impaired the responsiveness to Zta (Fig. 4B), indicating that all of them are essential for the full Zta-triggered activation of the MMP3 promoter. The AP-1 site at −216 seems to be most important since single mutation at this site abolished the promoter responsiveness to Zta. Furthermore, dual or triple mutation of these three AP-1 sites also completely blocked the Zta-induced transactivation. Therefore, Zta activates the MMP3 promoter through multiple AP-1 elements.

### The DNA-binding Domain of Zta is Required for Recruitment to the MMP3 Promoter and for Induction of MMP3 Expression

**Figure 5:**
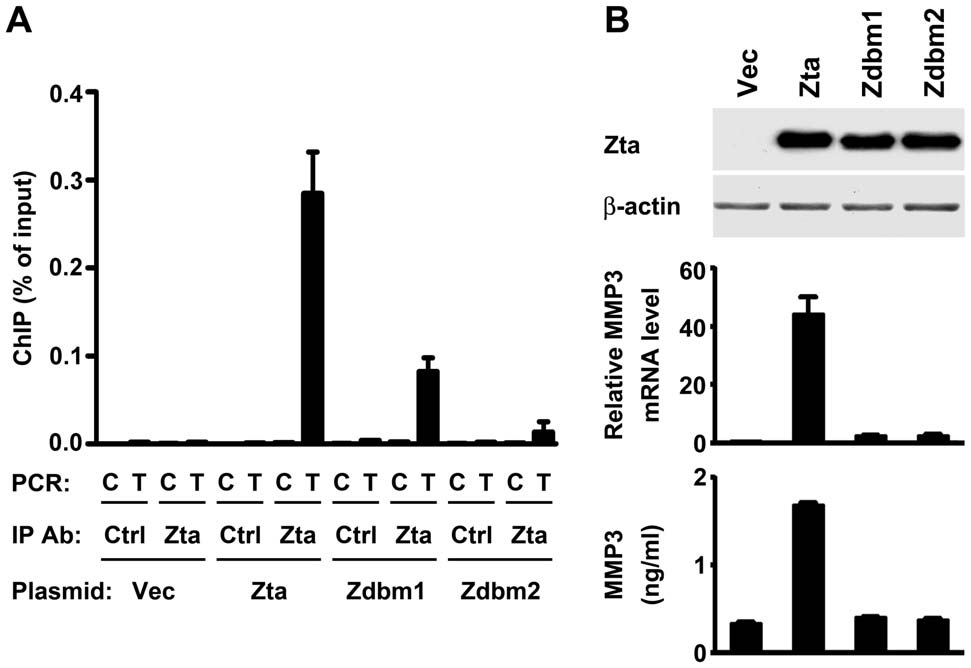
The DNA-binding domain of Zta is required for induction of MMP3 expression. (**A**) NPC cells were transfected with a vector plasmid (Vec) or plasmids expressing wild-type Zta or the DNA binding-defective mutants (Zdbm1 and Zdbm2) and then subjected to a ChIP assay. The antibodies used for immunoprecipitation (IP Ab) included an anti-Zta antibody and a control antibody (Ctrl). In the immunoprecipitants, the target DNA region (T) spanning AP-1 elements of the MMP3 promoter and the control DNA region (C) distant from the AP-1 elements were quantified by using real-time PCR. The ChIP results were expressed as “percentage of input”. (**B**) Cells were transfected with a vector plasmid or plasmids expressing wild-type Zta, Zdbm1 or Zdbm2. Protein expression of Zta and β-actin was examined by an immunoblotting assay (upper panel). Expression levels of MMP3 mRNA were measured by quantitative RT-PCR (middle panel). Concentrations of MMP3 protein in the cell culture supernatants were quantified by using ELISA (lower panel).

A previous study indicates that Zta binds to the MMP9 promoter and that Zta mutants lacking the DNA-binding domain fail to induce MMP9 [4]. In this study, we tested whether the similar mechanism can also apply to Zta-mediated induction of MMP3. In a ChIP assay, wild-type Zta was recruited preferentially to the MMP3 promoter region spanning AP-1 elements but not to the control region distant from the AP-1 elements (Fig. 5A). In addition, we tested two Zta mutants, Zdbm1 and Zdbm2, of which the DNA binding domains are mutated [18], [34]. Both Zta mutants showed diminished recruitment to the MMP3 promoter (Fig. 5A) and failed to induce MMP3 expression at mRNA and protein levels (Fig. 5B). Therefore, similar to the mechanism of MMP9 induction, the DNA binding domain of Zta is also required for induction of MMP3.

### Zta-induced Cell Migration Requires MMP3 While Zta-induced Cell Invasion Requires Both MMP3 and MMP9

**Figure 6:**
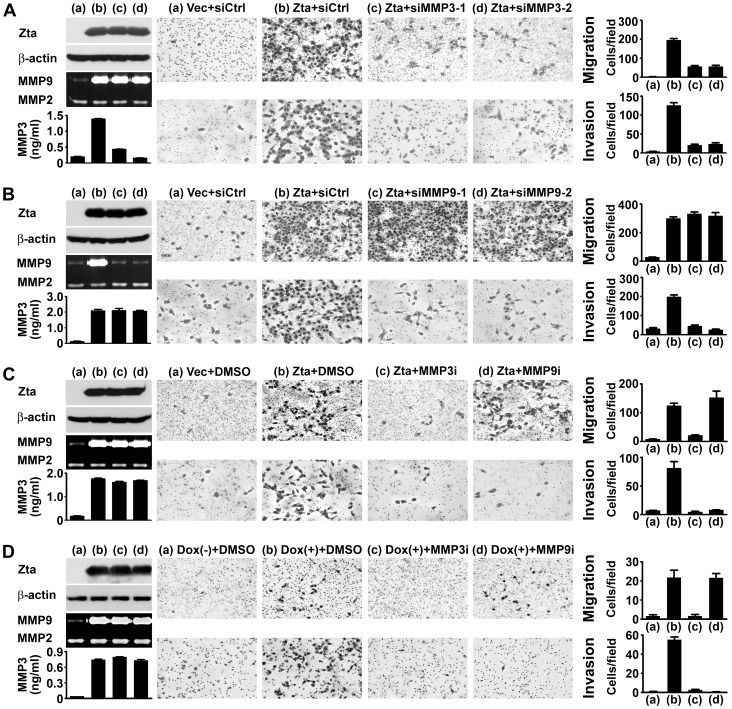
Zta-induced cell migration requires MMP3 while Zta-induced cell invasion requires both MMP3 and MMP9. (**A**) HONE-1 cells were cotransfected with a control siRNA (siCtrl) or MMP3-targeted siRNAs (siMMP3-1 and siMMP3-2) in combination with a vector plasmid (Vec) or a Zta-expressing plasmid (Zta). (**B**) HONE-1 cells were cotransfected with a control siRNA or MMP9-targeted siRNAs (siMMP9-1 and siMMP9-2) in combination with a vector plasmid or a Zta-expressing plasmid. (**C**) HONE-1 cells transfected with a vector plasmid or a Zta-expressing plasmid were treated with the solvent control DMSO, an MMP3 inhibitor (MMP3i), or an MMP9 inhibitor (MMP9i). (**D**) HONE-tetonZ cells with (+) or without (−) induction by doxycycline (Dox) were treated with the solvent control DMSO, an MMP3 inhibitor (MMP3i), or an MMP9 inhibitor (MMP9i). Protein expression of Zta and β-actin was examined by an immunoblotting assay. Production of MMP9 and MMP2 in the cell culture supernatants was detected by using a gelatin zymography assay. Concentrations of MMP3 in the cell culture supernatants were quantified by using ELISA. Cell migration was examined by using a transwell migration assay, and cell invasion in ECM was examined by using a Matrigel invasion assay. Shown are the microscopic observations of migrating or invading cells and the average numbers of the cells per field.

Next we examined the biologic effects of Zta-induced MMPs, testing whether MMP3 and MMP9 are involved in Zta-promoted migration and invasion of NPC cells *in vitro*. Compared with the vector control cells, Zta-transfected HONE-1 cells showed more migratory and invasive ability (Figs. 6A to 6C). We further used siRNAs to examine the contributions of MMP3 and MMP9 in this context. Knockdown of MMP3, which did not affect MMP9 induction, significantly reduced Zta-induced cell migration and invasion (Fig. 6A). On the other hand, knockdown of MMP9, which did not affect MMP3 induction, blocked cell invasion but had no effect on cell migration (Fig. 6B). These results suggest that Zta promotes cell migration in a MMP3-dependent, MMP9-independent manner, while Zta substantially promotes cell invasion through a mechanism requiring co-induction of MMP3 and MMP9. To confirm this hypothesis, we used specific MMP inhibitors that blocked the enzymatic activity but did not affect the protein levels of MMPs. Zta-induced cell migration was abolished by the MMP3 inhibitor but not by the MMP9 inhibitor, while Zta-induced cell invasion was blocked by either of the inhibitors (Fig. 6C). Similar results of the inhibitor treatment were also obtained when we used doxycycline-inducible, Zta-expressing HONE-tetonZ cells (Fig. 6D). Therefore, Zta-induced MMP3 and MMP9 may cause different effects on cell motility, but both of them are critical for Zta-promoted cell invasion.

### MMP3 Enhances Cell Migration and Synergizes with MMP9 to Promote Cell Invasion

**Figure 7:**
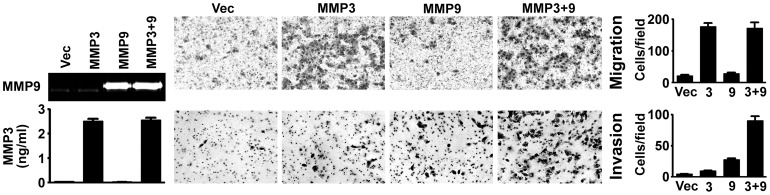
MMP3 enhances cell migration and synergizes with MMP9 to promote cell invasion. HONE-1 cells were transfected with a vector plasmid (Vec), a MMP3-expressing plasmid, or a MMP9-expressing plasmid, or cotransfected with both MMP3 and MMP9 plasmids (MMP3+9). production of MMP9 was examined by using a gelatin zymography assay, and production of MMP3 was examined by using ELISA. A transwell migration assay and a Matrigel invasion assay were performed. Shown are the microscopic observations of migrating or invading cells and the average numbers of the cells per field.

Next we wondered whether individual expression or co-expression of MMP3 and MMP9, in the absence of Zta, can result in the biologic effects similar to those induced by Zta. We examined motility and invasiveness of HONE-1 cells with ectopic expression of MMP3 and/or MMP9. MMP3 expressed alone sufficiently promoted cell migration (Fig. 7). MMP9 did not affect cell motility when expressed alone, and was unable to further enhance migration of the MMP3-expressing cells (Fig. 7, upper panel). Therefore, MMP3, not MMP9, functions as a promoter of NPC cell migration. On the other hand, though overexpression of MMP3 alone or MMP9 alone enhanced cell invasion weakly (2 to 4 folds compared with the vector control), the invasiveness was increased prominently (more than 13 folds) when MMP3 and MMP9 were co-expressed (Fig. 7, lower panel). This result indicates that MMP3 and MMP9 synergistically promote cell invasion, which may explain why Zta-induced cell invasion requires co-expression of both MMPs.

## Discussion

Extending from previous studies separately showing that Zta promotes cell migration/invasion and that Zta induces MMP expression [4], [22], this study provides integrated evidence demonstrating that Zta drives cell migration and invasion through induction of MMPs. Our new findings include that (1) MMP3, like MMP9 found previously, is induced by Zta; (2) MMP3 is required for Zta-induced cell migration but MMP9 is not; (3) both MMP3 and MMP9 contribute to Zta-induced cell invasion. Although these findings are based on *in vitro* experiments and we have not examined biopsy specimens, this study reveals a possible mechanism how Zta increases cell motility and invasiveness. It remains to be studied whether this mechanism is linked to a previous report showing that Zta expression correlates with neck metastasis of NPC [4].

Compared with a previous study about Zta-mediated induction of MMP9 [4], our present study indicates that Zta can also induce MMP3 expression and the induction mechanism is similar to that of MMP9. Both MMP3 and MMP9 are upregulated by Zta at a transcriptional level, and Zta activates the promoters of both MMPs through AP-1 elements. In addition, Zta binds to both promoters, and the DNA-binding domain of Zta is required for induction of both MMPs. Notably, all three AP-1 sites in the MMP3 promoter are essential for the responsiveness to Zta, suggesting that Zta needs multiple binding elements for the full transactivation. The MMP9 promoter also contains three AP-1 sites [36], but so far only one of them has been tested for Zta-mediated transactivation [4]. Multiple Zta-binding DNA elements are also found in the promoters of other Zta-regulated genes, including interleukin (IL)-10 [19], IL-8 [18], and early growth response-1 [20]. It is likely that cooperative interaction among adjacently DNA-bound Zta is important for transactivation of these promoters. A similar case is JunB, a member of AP-1 family proteins, which can activate the promoter containing multimeric AP-1 elements but not the promoter with only a single AP-1 site [39].

In addition to direct targeting of Zta to the promoters of MMP3 and MMP9, other mechanisms may also be involved in Zta-mediated induction of the MMPs. First, Zta may induce expression of other transcription factors to indirectly regulate the MMP promoters. For example, Zta has been previously shown to upregulate c-Fos and early growth response-1 [20], [40], two cellular transcription factors associated with induction of MMP3 and MMP9 [41]–[43]. Zta may also affect some cellular signaling pathways to regulate MMP expression. It has been reported that Zta can trigger activation of the ERK1/2 signaling pathway [20], which may be critical for induction of various MMPs [27], [36], [44]. Alternatively, MMP expression may be modulated by some Zta-induced secreted factors in an autocrine or paracrine manner. IL-8 and prostaglandin E_2_, two secreted factors that can be augmented by Zta [18], [37], have been shown to upregulate MMP3 and MMP9 [45], [46]. Besides, our unpublished data indicated that Zta can induce heparin-binding epidermal growth factor, which is another secreted factor potentially linked to induction of both MMPs [47].

Considering that many promoters of MMP genes contain similar *cis*-regulatory elements, multiple MMPs can be co-regulated by some common inducers or mechanisms [47], [48]. However, according to our MMP array analysis, only MMP3 and MMP9 can be prominently upregulated by Zta. Although the promoters of MMP1 and MMP10 genes contain AP-1 elements [49], Zta does not induce these two MMPs in our study, in contrast to a previous study showing Zta-induced MMP1 expression in a keratinocyte cell line [22]. Therefore, in addition to existence of AP-1 elements in the MMP promoters, there must be other cell-specific requirements determining MMP induction by Zta. One possibility is the DNA methylation status of MMP promoters, as Zta preferentially binds to and activates methylated promoters [50], [51]. In line with this notion, methylation status of MMP promoters has been reported to be cell-specific, and the epigenetic status affects MMP expression [52], [53]. On the other hand, AP-1 proteins may cooperate with different cofactors for regulation of individual MMP genes. For example, AP-1-mediated induction of MMP1 requires ETS1 [54] while the induction of MMP9 depends on cooperation with STAT3 [55]. Being an AP-1-like b-Zip transcription factor, Zta may also interact with some cell-specific cofactors that result in the differential MMP induction by Zta.

Co-expression of multiple MMPs has been detected in tumor tissues and associated with cancer progression [56], [57]. However, it remains unclear whether the co-expressed MMPs exert independent, redundant, or synergistic effects on cancer progression. In this study, MMP3 and MMP9 differentially affect cell migration. Blocking or overexpression of MMP9 did not influence cell migration, indicating that MMP9 has minimal effect on cell migration. By contrast, knockdown or inhibition of MMP3 blocked Zta-induced cell migration and ectopic expression of MMP3 alone sufficiently increased cell motility, indicating that MMP3 plays a critical role therein. MMP3 may promote cell migration in several ways. For example, MMP3 may cleave E-cadherin to facilitate cell disassociation, increase cell motility, and trigger epithelial-to-mesenchymal transition [58]. MMP3 may also induce production of keratinocyte growth factor [58], a secreted protein associated with enhanced cell migration [59]. Further studies are required to identify the mechanisms how Zta-induced MMP3 promotes cell migration.

On the other hand, this study indicates that Zta-induced MMP3 and MMP9 synergistically drive cell invasion. MMP9 is a well-documented MMP that digests ECM and facilitates cell invasion. Interestingly, our study indicates that MMP3 also essentially contributes to cell invasiveness in the presence of MMP9. Knockdown or inhibition of MMP3 suppressed Zta-induced cell invasiveness in ECM. In addition, although ectopic expression of MMP3 alone barely influenced cell invasion, it significantly increased cell invasiveness when MMP9 was co-expressed. The synergy of MMP3 and MMP9 may be explained by several potential mechanisms. First, our data show that Zta-induced MMP3 promotes cell migration, which may be critical and prerequisite for subsequent MMP9-induced cell invasion. Second, MMP3 can be an upstream activator of other MMPs including MMP9 [49], [58], [60], so MMP3 may enhance enzymatic activity of MMP9 to promote invasion. Meanwhile, cell invasion can be augmented when MMP9 is recruited by CD44 to the invasive front of cells [24], [61]. Considering that MMP3 can induces keratinocyte growth factor [58], which upregulates CD44 expression [62], thus MMP3 may indirectly facilitate surface recruitment of MMP9 to promote cell invasion.

Through MMP3 and MMP9, Zta may regulate other pathogenic effects in addition to cell migration and invasion. Based on the broad substrates of MMP3 and MMP9, their potential effects may be involved in multiple steps of cancer progression, including cell survival, proliferation, epithelial-to-mesenchymal transition, migration, angiogenesis, immunosuppression, and metastasis [24], [63], [64]. Notably, MMP3 and/or MMP9 can also be upregulated by other EBV proteins, such as latent membrane protein-1 and -2A [36], [65], [66]. It is likely that EBV has evolved multiple ways for induction of MMPs, probably explaining the prevalent detection of MMPs in NPC tumor tissues [22], [65]. Thus EBV-induced MMP3 and MMP9 may work individually or cooperatively to promote cancer progression. Interestingly, it has been reported that hepatitis B virus X protein can also induce MMP3 and MMP9, resulting in enhanced cell motility and invasiveness [44], [67]. MMP3-specific inhibitors can abolish the X protein-induced cell migration [67] and can also block Zta-induced cell migration and invasion in our study. Since MMP3 may function as a key activator of other MMPs [49], blocking this critical protease can be a potential approach to treat EBV-induced, MMP-mediated pathogenesis.

## References

[1] Chan AT, Teo PM, Johnson PJ (2002) Nasopharyngeal carcinoma. Ann Oncol 13: 1007–1015. 12176778 10.1093/annonc/mdf179

[2] Raab-Traub N (2002) Epstein-Barr virus in the pathogenesis of NPC. Semin Cancer Biol 12: 431–441. 12450729 10.1016/s1044579x0200086x

[3] Cochet C, Martel-Renoir D, Grunewald V, Bosq J, Cochet G, et al (1993) Expression of the Epstein-Barr virus immediate early gene, BZLF1, in nasopharyngeal carcinoma tumor cells. Virology 197: 358–365. 8212572 10.1006/viro.1993.1597

[4] Yoshizaki T, Sato H, Murono S, Pagano JS, Furukawa M (1999) Matrix metalloproteinase 9 is induced by the Epstein-Barr virus BZLF1 transactivator. Clin Exp Metastasis 17: 431–436. 10651310 10.1023/a:1006699003525

[5] Martel-Renoir D, Grunewald V, Touitou R, Schwaab G, Joab I (1995) Qualitative analysis of the expression of Epstein-Barr virus lytic genes in nasopharyngeal carcinoma biopsies. J Gen Virol 76: 1401–1408. 7782768 10.1099/0022-1317-76-6-1401

[6] Chien YC, Chen JY, Liu MY, Yang HI, Hsu MM, et al (2001) Serologic markers of Epstein-Barr virus infection and nasopharyngeal carcinoma in Taiwanese men. N Engl J Med 345: 1877–1882. 11756578 10.1056/NEJMoa011610

[7] de-Vathaire F, Sancho-Garnier H, de-The H, Pieddeloup C, Schwaab G, et al (1988) Prognostic value of EBV markers in the clinical management of nasopharyngeal carcinoma (NPC): a multicenter follow-up study. Int J Cancer 42: 176–181. 2841245 10.1002/ijc.2910420206

[8] Henle W, Ho JH, Henle G, Chau JC, Kwan HC (1977) Nasopharyngeal carcinoma: significance of changes in Epstein-Barr virus-related antibody patterns following therapy. Int J Cancer 20: 663–672. 200569 10.1002/ijc.2910200504

[9] Yip TT, Ngan RK, Lau WH, Poon YF, Joab I, et al (1994) A possible prognostic role of immunoglobulin-G antibody against recombinant Epstein-Barr virus BZLF-1 transactivator protein ZEBRA in patients with nasopharyngeal carcinoma. Cancer 74: 2414–2424. 7922994 10.1002/1097-0142(19941101)74:9<2414::aid-cncr2820740905>3.0.co;2-8

[10] Bouvier G, Hergenhahn M, Polack A, Bornkamm GW, de The G, et al (1995) Characterization of macromolecular lignins as Epstein-Barr virus inducer in foodstuff associated with nasopharyngeal carcinoma risk. Carcinogenesis 16: 1879–1885. 7634418 10.1093/carcin/16.8.1879

[11] Shao YM, Poirier S, Ohshima H, Malaveille C, Zeng Y, et al (1988) Epstein-Barr virus activation in Raji cells by extracts of preserved food from high risk areas for nasopharyngeal carcinoma. Carcinogenesis 9: 1455–1457. 2841048 10.1093/carcin/9.8.1455

[12] Fang CY, Lee CH, Wu CC, Chang YT, Yu SL, et al (2009) Recurrent chemical reactivations of EBV promotes genome instability and enhances tumor progression of nasopharyngeal carcinoma cells. Int J Cancer 124: 2016–2025. 19132751 10.1002/ijc.24179

[13] Huang SY, Fang CY, Tsai CH, Chang Y, Takada K, et al (2010) N-methyl-N’-nitro-N-nitrosoguanidine induces and cooperates with 12-O-tetradecanoylphorbol-1,3-acetate/sodium butyrate to enhance Epstein-Barr virus reactivation and genome instability in nasopharyngeal carcinoma cells. Chem Biol Interact 188: 623–634. 20869957 10.1016/j.cbi.2010.09.020

[14] Rooney CM, Rowe DT, Ragot T, Farrell PJ (1989) The spliced BZLF1 gene of Epstein-Barr virus (EBV) transactivates an early EBV promoter and induces the virus productive cycle. J Virol 63: 3109–3116. 2542618 10.1128/jvi.63.7.3109-3116.1989 PMC250868

[15] Grogan E, Jenson H, Countryman J, Heston L, Gradoville L, et al (1987) Transfection of a rearranged viral DNA fragment, WZhet, stably converts latent Epstein-Barr viral infection to productive infection in lymphoid cells. Proc Natl Acad Sci U S A 84: 1332–1336. 3029778 10.1073/pnas.84.5.1332 PMC304422

[16] Kouzarides T, Packham G, Cook A, Farrell PJ (1991) The BZLF1 protein of EBV has a coiled coil dimerisation domain without a heptad leucine repeat but with homology to the C/EBP leucine zipper. Oncogene 6: 195–204. 1847997

[17] Holley-Guthrie EA, Quinlivan EB, Mar EC, Kenney S (1990) The Epstein-Barr virus (EBV) BMRF1 promoter for early antigen (EA-D) is regulated by the EBV transactivators, BRLF1 and BZLF1, in a cell-specific manner. J Virol 64: 3753–3759. 2164595 10.1128/jvi.64.8.3753-3759.1990 PMC249670

[18] Hsu M, Wu SY, Chang SS, Su IJ, Tsai CH, et al (2008) Epstein-Barr virus lytic transactivator Zta enhances chemotactic activity through induction of interleukin-8 in nasopharyngeal carcinoma cells. J Virol 82: 3679–3688. 18234802 10.1128/JVI.02301-07 PMC2268478

[19] Mahot S, Sergeant A, Drouet E, Gruffat H (2003) A novel function for the Epstein-Barr virus transcription factor EB1/Zta: induction of transcription of the hIL-10 gene. J Gen Virol 84: 965–974. 12655098 10.1099/vir.0.18845-0

[20] Chang Y, Lee HH, Chen YT, Lu J, Wu SY, et al (2006) Induction of the early growth response 1 gene by Epstein-Barr virus lytic transactivator Zta. J Virol 80: 7748–7755. 16840354 10.1128/JVI.02608-05 PMC1563714

[21] Joab I, Nicolas JC, Schwaab G, de-The G, Clausse B, et al (1991) Detection of anti-Epstein-Barr-virus transactivator (ZEBRA) antibodies in sera from patients with nasopharyngeal carcinoma. Int J Cancer 48: 647–649. 1649137 10.1002/ijc.2910480503

[22] Lu J, Chua HH, Chen SY, Chen JY, Tsai CH (2003) Regulation of matrix metalloproteinase-1 by Epstein-Barr virus proteins. Cancer Res 63: 256–262. 12517806

[23] Kessenbrock K, Plaks V, Werb Z (2010) Matrix metalloproteinases: regulators of the tumor microenvironment. Cell 141: 52–67. 20371345 10.1016/j.cell.2010.03.015 PMC2862057

[24] Egeblad M, Werb Z (2002) New functions for the matrix metalloproteinases in cancer progression. Nat Rev Cancer 2: 161–174. 11990853 10.1038/nrc745

[25] Johnson JL, Pillai S, Pernazza D, Sebti SM, Lawrence NJ, et al (2012) Regulation of matrix metalloproteinase genes by E2F transcription factors: Rb-Raf-1 interaction as a novel target for metastatic disease. Cancer Res 72: 516–526. 22086850 10.1158/0008-5472.CAN-11-2647 PMC3261351

[26] Hung WC, Tseng WL, Shiea J, Chang HC (2010) Skp2 overexpression increases the expression of MMP-2 and MMP-9 and invasion of lung cancer cells. Cancer Lett 288: 156–161. 19625121 10.1016/j.canlet.2009.06.032

[27] Luo Y, Liang F, Zhang ZY (2009) PRL1 promotes cell migration and invasion by increasing MMP2 and MMP9 expression through Src and ERK1/2 pathways. Biochemistry 48: 1838–1846. 19199380 10.1021/bi8020789 PMC2765538

[28] Glaser R, Zhang HY, Yao KT, Zhu HC, Wang FX, et al (1989) Two epithelial tumor cell lines (HNE-1 and HONE-1) latently infected with Epstein-Barr virus that were derived from nasopharyngeal carcinomas. Proc Natl Acad Sci U S A 86: 9524–9528. 2556716 10.1073/pnas.86.23.9524 PMC298529

[29] Lu J, Chen SY, Chua HH, Liu YS, Huang YT, et al (2000) Upregulation of tyrosine kinase TKT by the Epstein-Barr virus transactivator Zta. J Virol 74: 7391–7399. 10906192 10.1128/jvi.74.16.7391-7399.2000 PMC112259

[30] Huang SY, Hsieh MJ, Chen CY, Chen YJ, Chen JY, et al (2012) Epstein-Barr virus Rta-mediated transactivation of p21 and 14–3-3σ arrests cells at the G1/S transition by reducing cyclin E/CDK2 activity. J Gen Virol 93: 139–149. 21918011 10.1099/vir.0.034405-0

[31] Maruo S, Yang L, Takada K (2001) Roles of Epstein-Barr virus glycoproteins gp350 and gp25 in the infection of human epithelial cells. J Gen Virol 82: 2373–2383. 11562531 10.1099/0022-1317-82-10-2373

[32] Rheinwald JG, Beckett MA (1981) Tumorigenic keratinocyte lines requiring anchorage and fibroblast support cultures from human squamous cell carcinomas. Cancer Res 41: 1657–1663. 7214336

[33] Barranco SC, Townsend CM Jr, Casartelli C, Macik BG, Burger NL, et al (1983) Establishment and characterization of an in vitro model system for human adenocarcinoma of the stomach. Cancer Res 43: 1703–1709. 6831414

[34] Flemington EK, Lytle JP, Cayrol C, Borras AM, Speck SH (1994) DNA-binding-defective mutants of the Epstein-Barr virus lytic switch activator Zta transactivate with altered specificities. Mol Cell Biol 14: 3041–3052. 8164660 10.1128/mcb.14.5.3041-3052.1994 PMC358672

[35] Shih WL, Liao MH, Yu FL, Lin PY, Hsu HY, et al (2008) AMF/PGI transactivates the MMP-3 gene through the activation of Src-RhoA-phosphatidylinositol 3-kinase signaling to induce hepatoma cell migration. Cancer Lett 270: 202–217. 18571835 10.1016/j.canlet.2008.05.005

[36] Lan YY, Hsiao JR, Chang KC, Chang JS, Chen CW, et al (2012) Epstein-Barr virus latent membrane protein 2A promotes invasion of nasopharyngeal carcinoma cells through ERK/Fra-1-mediated induction of matrix metalloproteinase 9. J Virol 86: 6656–6667. 22514348 10.1128/JVI.00174-12 PMC3393536

[37] Lee CH, Yeh TH, Lai HC, Wu SY, Su IJ, et al (2011) Epstein-Barr virus Zta-induced immunomodulators from nasopharyngeal carcinoma cells upregulate interleukin-10 production from monocytes. J Virol 85: 7333–7342. 21543473 10.1128/JVI.00182-11 PMC3126557

[38] Ragoczy T, Heston L, Miller G (1998) The Epstein-Barr virus Rta protein activates lytic cycle genes and can disrupt latency in B lymphocytes. J Virol 72: 7978–7984. 9733836 10.1128/jvi.72.10.7978-7984.1998 PMC110133

[39] Chiu R, Angel P, Karin M (1989) Jun-B differs in its biological properties from, and is a negative regulator of, c-Jun. Cell 59: 979–986. 2513128 10.1016/0092-8674(89)90754-x

[40] Flemington E, Speck SH (1990) Epstein-Barr virus BZLF1 trans activator induces the promoter of a cellular cognate gene, c-fos. J Virol 64: 4549–4552. 2166830 10.1128/jvi.64.9.4549-4552.1990 PMC247926

[41] Fauquier L, Duboe C, Jore C, Trouche D, Vandel L (2008) Dual role of the arginine methyltransferase CARM1 in the regulation of c-Fos target genes. FASEB J 22: 3337–3347. 18511550 10.1096/fj.07-104604

[42] Chandrasekar B, Mummidi S, Mahimainathan L, Patel DN, Bailey SR, et al (2006) Interleukin-18-induced human coronary artery smooth muscle cell migration is dependent on NF-kappaB- and AP-1-mediated matrix metalloproteinase-9 expression and is inhibited by atorvastatin. J Biol Chem 281: 15099–15109. 16554298 10.1074/jbc.M600200200

[43] Shin SY, Kim JH, Baker A, Lim Y, Lee YH (2010) Transcription factor Egr-1 is essential for maximal matrix metalloproteinase-9 transcription by tumor necrosis factor alpha. Mol Cancer Res 8: 507–519. 20332214 10.1158/1541-7786.MCR-09-0454

[44] Chung TW, Lee YC, Kim CH (2004) Hepatitis B viral HBx induces matrix metalloproteinase-9 gene expression through activation of ERK and PI-3K/AKT pathways: involvement of invasive potential. FASEB J 18: 1123–1125. 15132991 10.1096/fj.03-1429fje

[45] Inoue K, Slaton JW, Kim SJ, Perrotte P, Eve BY, et al (2000) Interleukin 8 expression regulates tumorigenicity and metastasis in human bladder cancer. Cancer Res 60: 2290–2299. 10786697

[46] Bu X, Zhao C, Dai X (2011) Involvement of COX-2/PGE_2_ pathway in the upregulation of MMP-9 expression in pancreatic cancer. Gastroenterol Res Pract 2011: 214269. 21760774 10.1155/2011/214269 PMC3132487

[47] Ongusaha PP, Kwak JC, Zwible AJ, Macip S, Higashiyama S, et al (2004) HB-EGF is a potent inducer of tumor growth and angiogenesis. Cancer Res 64: 5283–5290. 15289334 10.1158/0008-5472.CAN-04-0925

[48] Belguise K, Kersual N, Galtier F, Chalbos D (2005) FRA-1 expression level regulates proliferation and invasiveness of breast cancer cells. Oncogene 24: 1434–1444. 15608675 10.1038/sj.onc.1208312

[49] Chakraborti S, Mandal M, Das S, Mandal A, Chakraborti T (2003) Regulation of matrix metalloproteinases: an overview. Mol Cell Biochem 253: 269–285. 14619979 10.1023/a:1026028303196

[50] Dickerson SJ, Xing Y, Robinson AR, Seaman WT, Gruffat H, et al (2009) Methylation-dependent binding of the epstein-barr virus BZLF1 protein to viral promoters. PLoS Pathog 5: e1000356. 19325883 10.1371/journal.ppat.1000356 PMC2654727

[51] Bhende PM, Seaman WT, Delecluse HJ, Kenney SC (2004) The EBV lytic switch protein, Z, preferentially binds to and activates the methylated viral genome. Nat Genet 36: 1099–1104. 15361873 10.1038/ng1424

[52] Couillard J, Demers M, Lavoie G, St-Pierre Y (2006) The role of DNA hypomethylation in the control of stromelysin gene expression. Biochem Biophys Res Commun 342: 1233–1239. 16516860 10.1016/j.bbrc.2006.02.068

[53] Chicoine E, Esteve PO, Robledo O, Van Themsche C, Potworowski EF, et al (2002) Evidence for the role of promoter methylation in the regulation of MMP-9 gene expression. Biochem Biophys Res Commun 297: 765–772. 12359218 10.1016/s0006-291x(02)02283-0

[54] Westermarck J, Seth A, Kahari VM (1997) Differential regulation of interstitial collagenase (MMP-1) gene expression by ETS transcription factors. Oncogene 14: 2651–2660. 9178763 10.1038/sj.onc.1201111

[55] Song Y, Qian L, Song S, Chen L, Zhang Y, et al (2008) Fra-1 and Stat3 synergistically regulate activation of human MMP-9 gene. Mol Immunol 45: 137–143. 17572495 10.1016/j.molimm.2007.04.031

[56] Saez E, Rutberg SE, Mueller E, Oppenheim H, Smoluk J, et al (1995) c-fos is required for malignant progression of skin tumors. Cell 82: 721–732. 7545543 10.1016/0092-8674(95)90469-7

[57] Mendes O, Kim HT, Stoica G (2005) Expression of MMP2, MMP9 and MMP3 in breast cancer brain metastasis in a rat model. Clin Exp Metastasis 22: 237–246. 16158251 10.1007/s10585-005-8115-6

[58] Lochter A, Galosy S, Muschler J, Freedman N, Werb Z, et al (1997) Matrix metalloproteinase stromelysin-1 triggers a cascade of molecular alterations that leads to stable epithelial-to-mesenchymal conversion and a premalignant phenotype in mammary epithelial cells. J Cell Biol 139: 1861–1872. 9412478 10.1083/jcb.139.7.1861 PMC2132651

[59] Zang XP, Bullen EC, Manjeshwar S, Jupe ER, Howard EW, et al (2006) Enhanced motility of KGF-transfected breast cancer cells. Anticancer Res 26: 961–966. 16619493

[60] Ogata Y, Enghild JJ, Nagase H (1992) Matrix metalloproteinase 3 (stromelysin) activates the precursor for the human matrix metalloproteinase 9. J Biol Chem 267: 3581–3584. 1371271

[61] Yu Q, Stamenkovic I (1999) Localization of matrix metalloproteinase 9 to the cell surface provides a mechanism for CD44-mediated tumor invasion. Genes Dev 13: 35–48. 9887098 10.1101/gad.13.1.35 PMC316376

[62] Karvinen S, Pasonen-Seppanen S, Hyttinen JM, Pienimaki JP, Torronen K, et al (2003) Keratinocyte growth factor stimulates migration and hyaluronan synthesis in the epidermis by activation of keratinocyte hyaluronan synthases 2 and 3. J Biol Chem 278: 49495–49504. 14506240 10.1074/jbc.M310445200

[63] Sheu BC, Hsu SM, Ho HN, Lien HC, Huang SC, et al (2001) A novel role of metalloproteinase in cancer-mediated immunosuppression. Cancer Res 61: 237–242. 11196168

[64] Bergers G, Brekken R, McMahon G, Vu TH, Itoh T, et al (2000) Matrix metalloproteinase-9 triggers the angiogenic switch during carcinogenesis. Nat Cell Biol 2: 737–744. 11025665 10.1038/35036374 PMC2852586

[65] Lee DC, Chua DT, Wei WI, Sham JS, Lau AS (2007) Induction of matrix metalloproteinases by Epstein-Barr virus latent membrane protein 1 isolated from nasopharyngeal carcinoma. Biomed Pharmacother 61: 520–526. 17913445 10.1016/j.biopha.2007.08.007

[66] Yoshizaki T, Sato H, Furukawa M, Pagano JS (1998) The expression of matrix metalloproteinase 9 is enhanced by Epstein-Barr virus latent membrane protein 1. Proc Natl Acad Sci U S A 95: 3621–3626. 9520415 10.1073/pnas.95.7.3621 PMC19885

[67] Yu FL, Liu HJ, Lee JW, Liao MH, Shih WL (2005) Hepatitis B virus X protein promotes cell migration by inducing matrix metalloproteinase-3. J Hepatol 42: 520–527. 15763339 10.1016/j.jhep.2004.11.031

